# Vertical smooth pursuit as a diagnostic marker of traumatic brain injury

**DOI:** 10.2217/cnc-2019-0013

**Published:** 2020-01-14

**Authors:** Melissa Hunfalvay, Claire-Marie Roberts, Nicholas P Murray, Ankur Tyagi, Kyle W Barclay, Takumi Bolte, Hannah Kelly, Frederick R Carrick

**Affiliations:** 1 RightEye LLC, 7979 Old Georgetown Rd, Suite 801, Bethesda, MD 20814, USA; 2 Department of Psychology, Senior Research Fellow, University of the West of England, Coldharbour Lane, Bristol, BS16 1QY, England; 3 Department of Kinesiology, East Carolina University, Minges Coliseum 166, Greensville, NC 27858, USA; 4 RightEye LLC, 7979 Old Georgetown Rd, Suite 801, Bethesda, MD 20814, USA; 5 Case Western Reserve University, 10501 Streamview Court, Potomac, MD 20854, USA; 6 RightEye LLC, 7979 Old Georgetown Rd, Suite 801, Bethesda, MD 20814, USA; 7 Emory University, 201 Dowman Dr, Atlanta, GA 30322, USA; 8 Centre for Mental Health Research in association with University of Cambridge, Cambridge UK; 9 Department of Neurology, University of Central Florida College of Medicine, Orlando, FL 32827, USA; 10 MGH Institute for Health Professions, Boston, MA, USA; 11 Carrick Institute, Cape Canaveral, FL 32920, USA

**Keywords:** concussion, eye tracking, TBI, vertical smooth pursuit

## Abstract

**Aim:**

Neural deficits were measured via the eye tracking of vertical smooth pursuit (VSP) as markers of traumatic brain injury (TBI). The present study evaluated the ability of the eye tracking tests to differentiate between different levels of TBI severity and healthy controls.

**Methodology:**

Ninety-two individuals divided into four groups (those with mild, moderate or severe TBI and healthy controls) participated in a computerized test of VSP eye movement using a remote eye tracker.

**Results:**

The VSP eye tracking test was able to distinguish between severe and moderate levels of TBI but unable to detect differences in the performance of participants with mild TBI and healthy controls.

**Conclusion:**

The eye-tracking technology used to measure VSP eye movements is able to provide a timely and objective method of differentiating between individuals with moderate and severe levels of TBI.

Worldwide, traumatic brain injury (TBI) contributes to death and disability more than any other traumatic insult [1]. In order to increase the likelihood of more positive outcomes for TBI patients, the key is early detection and diagnosis. While many traditional diagnostic approaches for TBI rely heavily on subjectivity, the objective measurement of eye movements can detect neural dysfunction [2] associated with head injuries [3]. Oculomotor behavior assessment for clinical purposes encompasses the following eye movement types: fixations, saccades and smooth pursuits [4]. Fixations involve maintaining gaze on a single location of high visual acuity [5]. Saccades quickly move the fovea between fixation points [6], and smooth pursuits allow an individual to track a moving object closely [7,8,9]. Different brain areas are involved in these different types of eye movement; for example, the smooth pursuit pathway in primates begins with M retinal ganglion cells, neurons that process motion information and transmit signals from the lateral geniculate nucleus to V1/striate cortex [10]. Visual information then flows from striate to extrastriate areas that project to the brainstem and other cortical and subcortical areas, including the frontal eye field (FEF) and supplementary eye field in the frontal lobe, middle temporal and medial superior temporal areas, the intraparietal and posterior parietal cortices, and parts of the cerebellum. Furthermore, smooth pursuit eye movements activate certain brain regions based on the direction of visual stimuli. For horizontal stimuli, the middle temporal and medial superior temporal areas and the FEF each project to the dorsolateral pontine nuclei, which sends projections to the cerebellum. Cerebellar structures then project to the medial vestibular nucleus in the brainstem. The pathway for vertical pursuits resembles that for horizontal, except it includes the rostral nucleus reticularis tegmenti pontis instead of the dorsolateral pontine nuclei. Also, instead of the medial vestibular nucleus, the vertical circuitry involves the y-group nucleus and its projections [10]. In tests of circular smooth pursuits where subjects track a moving target on a curved path, functional MRI studies have shown bilateral activation of the visual cortex, areas of the parietal cortex, area middle temporal and some activation in the FEF [11]. Examining the neurocircuitry regulating oculomotor behavior is valuable to understanding both normal functioning and pathophysiology, since various injuries and neurological diseases can impair smooth pursuit performance, including TBI [12].

TBIs are broadly categorized as mild, moderate or severe depending on a patient’s Glasgow Coma Score (GCS) [1]. The GCS is the most common scoring system used to evaluate a patient’s level of consciousness on a 3–15 point scale. The GCS rates a patient’s best motor response (out of six points), best verbal response (out of five points) and eye-opening ability (out of four points) [13]. The final GCS is a sum of the scores associated with each function (motor response, verbal response and eye-opening ability). Mild TBI (mTBI) is diagnosed when an individual scores between 13 and 15 on the GCS. Moderate TBI is classified by a GCS score of between 9 and 12, and severe TBI is associated with a GCS score of between 3 and 8 [1]. The most common severity of TBI is mTBI, which often occurs as a result of concussions or brain injuries from blows to the head or body that induce neurological symptoms [1,14]. In general, detecting and diagnosing concussion is a challenging process as the injury cannot be seen. Therefore, common approaches to concussion testing involve asking the affected individual to self-report their symptoms, as well as undertake neuropsychological testing [15]. Although this approach to testing is quick and cost-effective, the heavy subjectivity of the test interpretation and likelihood of under reporting devalues its efficacy. Additionally, the long-term validity of this approach to testing is questionable as both cognitive and visual deficits associated with concussion can endure long after the initial symptoms lessen [15]. One of the most common concussion testing approaches in sport involves the Sport Concussion Assessment Tool (SCAT-5) and the Child SCAT-5, which each comprise a combination of GCS scoring, an evaluation of cognitive and sensorimotor functions, a physical examination, the Standardized Assessment of Concussion and the Balance Error Scoring System scales [16]. The SCAT-5 approach to concussion detection does not specifically test vision [17]. Similarly, there is a new mobile phone app-based technology called the Defense Automated Neurobehavioral Assessment (DANA) that hosts cognitive and psychological tests with the intention of swift and reliable detection of TBI; however, individual motivation is likely to adversely influence the individual response [18,19,20]. In response to the lack of objectivity in the aforementioned tests, there are specific measures of evaluation of saccades – for example in the King-Devick Test, but this does not examine other eye movements that are often diminished after TBI [16,17]. Additionally, and with a more wide-ranging approach to evaluating oculomotor behavior, the Vestibular/Ocular Motor Screening method measures a combination of saccades, smooth pursuits, fixations, convergence and the vestibular–ocular reflex, and has been shown to be able to differentiate athletes with mTBI from healthy controls [15]. The measurement of these oculomotor variables is, however, achieved via a combination of symptom self-report, which is heavily open to subjectivity, bias and under reporting, and clinician observation of gross eye movements [17]. One notable exception to the variety of tests that fail to consider eye movements is OVRT or the Oculomotor Vestibular Reaction Time test battery. Though OVRT contains an impressive array of diagnostic tools, it does not consider vertical smooth pursuits (VSP) [21]. While it does consider horizontal smooth pursuits, they do not activate the same brain regions as VSP, and thus information about a particular brain region is not considered in its testing [10,11].

As a result of the shortcomings of many of the test options available for concussion and TBI detection, it is important to uncover additional, objective methods of measurement that may help with diagnostic decision making. One option for consideration is an extension of the use of eye-tracking technology, which is capable of detecting a number of different types of eye movement several times per second [7]. This technology is able to detect damage to neural circuitry as a result of TBI, with the production of sensitive quantifiable data that can be added to other TBI screening methods [22,23]. As the widespread cortico-cerebellar circuits involved in smooth pursuits are easily compromised after TBI, smooth pursuit deficits are important to measure [23]. Two metrics for smooth pursuits include variance and smooth pursuit percentage (SP%). Variance measures the deviance of a gaze path from the ideal path of a stimulus; a smaller spread of gaze positions around the target path indicates better accuracy [22]. SP% defines the amount of time spent performing a smooth pursuit with appropriate dispersion and velocity throughout a test. The velocity of the eye should match that of the moving stimulus to minimize position error [19]. Pursuit gain is a common velocity metric that calculates the ratio between eye and target velocity, where a larger gain implies better tracking ability [24].

Several studies have revealed deficits in the smooth pursuits of patients with concussion or post-concussion syndrome (PCS) [21,25,26]. These include greater lag during smooth pursuit tracking and lower gain of pursuit velocity in subjects with mTBI and PCS symptoms [19,27]. To investigate smooth pursuits, researchers often utilize circular tracking tests, a type of predictive visual tracking that necessitates attention and working memory [22]. As such cognitive processes require proper prefrontal cortex functioning and are vulnerable to damage from TBI [22], circular tracking tests can assess deficits in attention following TBI and may enhance the accuracy of traditional TBI screening [22,27]. Compared with controls, mTBI patients tracking an object in a circular path showed reduced target prediction and increased eye position error and variability; moreover, these impairments correlated with cognitive deficits [28,29]. Similarly, Maruta *et al.* found increased error and variability in gaze position with reduced smooth pursuit velocity in acute mTBI patients on a circular visual tracking task [24].

Although a few studies have specifically tested VSP performance following head trauma, there is evidence of TBI patients displaying impaired vertical pursuits. For example, subjects with symptomatic mTBI demonstrated smaller amplitude vertical pursuits [19] and reduced VSP velocity gain [19,24]. Furthermore, individuals with acute PCS symptoms exhibited a more rapid decrease in smooth pursuit velocity gain with increasing stimulus frequency [27]. This same patient group also showed reduced vertical gain as compared with horizontal gain for objects moving in a low-frequency range [27]. Further studies with larger sample sizes are necessary to increase the reliability and precision of results [27,28,29,30]. In addition, very few studies of oculomotor deficits following TBI have included tests of VSPs and no studies have concentrated exclusively on pursuits in the vertical direction. Given that different neural circuits control horizontal and vertical pursuits, it is important to thoroughly examine vertical pursuits in TBI patients for a more comprehensive evaluation of the different brain regions implicated in head injury [10,12]. Thus far, no studies have compared VSPs in healthy subjects to patients with different levels of TBI. The goal of this study, therefore, is to explore differences in VSPs measured by variance and SP% between individuals who have not had a TBI and patients diagnosed with TBI (either mild, moderate or severe).

## Methods

### Participants

For the data analysis, 92 participants were considered. Participants were between the ages of 11 and 79 years (mean [M] = 40.01, standard deviation [SD] = 16.87); 49 were males (53.26%) and 43 were females (46.74%). Of the 92 participants, 70.65% were white, 7.61% were Hispanic, 5.43% were Asians, 5.43% were black and 10.88% opted not to report ethnicity. The groups were matched by age (Table 1). All participants completed the RightEye Vertical Smooth Pursuit test (RightEye, LLC, MD, USA). There were 23 (25% of total participants in all categories of TBI severity) clinically verified participants in each of the following TBI severity levels: no-TBI, mTBI, moderate TBI and severe TBI. Participants were clinically verified by a board certified neurologist or neuro-optometrist, according to the medical diagnosis guidelines. The participants with TBI had sustained their head injuries within 30 days prior to the testing.

**Table 1.** Demographic data by age and gender.

Group (n)	Mean age (± SD)	Females	Males
No-TBI (23)	35.04 (16.84)	5	18
Mild (23)	39.74 (18.54)	12	11
Moderate (23)	42.26 (16.11)	10	13
Severe (23)	43.00 (15.80)	16	7

n: Number; SD: Standard deviation; TBI: Traumatic brain injury.

### Apparatus

**Figure 1.**
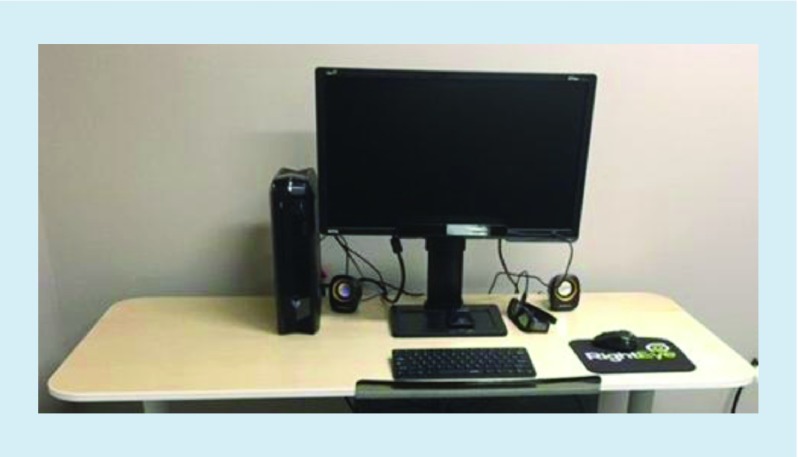
Testing apparatus.

The apparatus used in this study was identical to that reported in Hunfalvay *et al.* (2019) [31]. Please see Figure 1.

### Oculomotor Task

In the VSP test, participants were instructed to track a target stimulus – a white dot 0.2 degrees in diameter – at a rate of 25.13 degrees per second starting from the center of the screen. The dot then moved up and down in a sinusoidal way in the vertical plane in a straight line. For a participant to be considered on ‘target’, they were required to follow the stimuli within an error of 2.4 degrees. A participant could also be ahead or behind a stimulus and can still be labeled as ‘following’ if they are within an error of 4.8 degrees.

### Procedure

The research procedure used in the present study was identical to that reported by Hunfalvay *et al.* in 2019 [31] in the recruitment and prescreening section. To summarize, participants that qualified for the study were expected to pass a nine-point calibration sequence before they were asked to complete the eye tracking tests. The calibration sequence required participants to fixate one at a time on nine points displayed on the screen. The participants had to successfully fixate on at least eight out of the nine points on the screen to pass the calibration sequence. Written instructions on screen and animations were provided before each test to demonstrate appropriate behavior required in each of the tests.

### Data analysis

The differences in the groups (no-TBI, mild, moderate and severe TBI) were analyzed on clinically verified data. The comparison was evaluated using one-way univariate analysis of variance (ANOVA) on the smooth pursuit variance and SP% metrics. As the name suggests, smooth pursuit variance measures the variability in gaze while performing a vertical pursuit task. It is measured as SD (ml) in the average distance of each gaze sample point collected, from its expected ideal position. In other words, it explains the coherence in gaze when engaging in vertical pursuits. SP% is calculated as the participant’s eyes follow the target within a velocity range of the target. All such sample points are tallied to get the percentage over total test time. A *post-hoc* analysis was conducted using Tukey’s honestly significant difference (HSD) test, to determine the mean differences and their statistical significance, between each group. The α level was set at p < 0.05 for all statistical tests. In addition, receiving operating characteristic, area under the curve, sensitivity and specificity were calculated for a logistic regression to predict ‘no-TBI’ versus ‘all categories of TBI’ (mild, moderate and severe).

## Results

**Figure 2.**
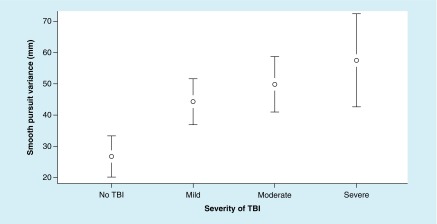
Mean values of smooth pursuit variance at each level of TBI severity, with 95% CI. For smooth pursuit variance metrics, a lower value is better. TBI: Traumatic brain injury.

The ANOVA for smooth pursuit variance metrics revealed significant differences between the groups. Smooth pursuit variance metrics resulted in a significant main effect, F (3, 88) = 4.52; p = 0.005, ω^2^ = 0.094. In addition, the Tukey’s HSD test demonstrated significant difference between moderate and severe TBI groups and the no-TBI group; however, there was no significant difference between mTBI and the no-TBI groups (Figure 2).

**Figure 3.**
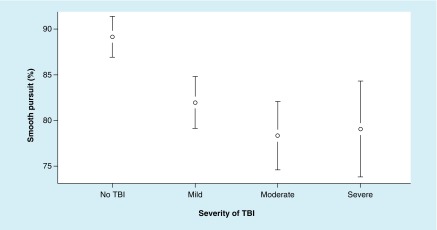
Mean values of smooth pursuit percentage at each level of TBI severity, with 95% CI. For smooth pursuit percentage metrics, a higher value is better. TBI: Traumatic brain injury.

The ANOVA for SP% metrics revealed significant differences between the groups; SP% metrics resulted in main effect, F (3, 88) = 3.80; p = 0.013, ω^2^ = 0.094. In addition, the Tukey’s HSD test demonstrated significant difference between moderate and severe TBI and the no-TBI groups; however, there was no significant difference between mTBI and the no-TBI groups (Figure 3).

**Figure 4.**
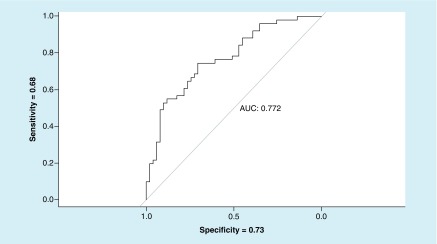
Receiving operating characteristic for vertical smooth pursuit (smooth pursuit variance and smooth pursuit [%]) – no-traumatic brain injury versus traumatic brain injury. AUC: Area under the curve.

The logistic regression model for smooth pursuit variance and SP% metrics revealed differentiated TBI and no-TBI groups. The resulting receiving operating characteristic curve produced an area under the curve value of 0.772 with sensitivity = 0.68 and specificity = 0.73 (Figure 4).

## Discussion

This is the first study to use eye tracking to compare VSP in healthy subjects to patients with different levels of clinically diagnosed TBI. Compromised smooth pursuits have been linked to TBI in several studies [21,25,26]. Smooth pursuits are a voluntary behavior and share a neural substrate with attention in the prefrontal cortex [23]; the frontal areas involved in both smooth pursuits and attention are connected to the cerebellum (also involved in smooth pursuits) via a nerve tract that is extremely susceptible to damage from TBI [22,23]. This relationship explains why many of the symptoms of TBI are associated with functions of the prefrontal cortex [22]. Because of this relationship, eye-tracking technology can serve as an indicator of neural dysfunction via eye movements. In this study, variance and SP% were the two metrics used to quantify the eye movements associated with smooth pursuit. Analysis of variance metrics revealed significant differences between the TBI groups and the control group. Results indicated that eye tracking can be used to distinguish between severe and moderate TBI and control groups, but not mild and control groups. Analysis of SP% metrics also yielded a significant difference between groups, along with a main effect. Similar to variance, SP% was able to distinguish severe and moderate groups from control groups but not the mTBI group. The VSP metrics proved least effective at distinguishing mTBI. This is surprising given that most research attention to date in this domain has focused on the use of VSP measurement to detect mTBI. Other metrics, such as horizontal and vertical saccades, are able to distinguish even mTBI from control groups; however, these eye movements are controlled by different brain regions [10,31]. Thus, even though VSPs might not have the highest capacity to differentiate, they are still highly relevant to a holistic model of TBI detection. This is especially true considering variance and SP% were the only two metrics being measured.

The four commonly accepted methods used to diagnose concussion are: diagnosis of concussion on the basis of the presence/duration of acute symptoms observed at the time of injury, including loss of consciousness, alteration of consciousness and/or post-traumatic amnesia; neurological; vestibular; and oculomotor [16]. Oculomotor can be further divided into types of eye movements including fixations, saccades and smooth pursuits [4]; each of these eye movements depend on different portions of the brain and thus test for different types of brain injury. Even among pursuits, vertical and horizontal pursuits differ in their neurological pathways [10,11]. The RightEye eye-tracking diagnostic tool at the center of this study is capable of measuring a number of metrics pertinent to TBI and has the potential to serve as a very useful adjunct to existing TBI symptoms detection methods. They are a multitude of tests currently used to diagnose concussions, all of which use some combination of the aforementioned diagnostic tools; however, most are deficient in at least one of the four categories [15]. For example, the current standard test for concussion in sport is the SCAT-5, which includes a physical exam, GCS and various cognitive and sensory-motor evaluations [16]. Though the SCAT-5 contains a slew of other tests including the Standard Assessment of Concussion and Balance Error Scoring System, it fails wholly to test vision [17]. This inconsistency is not unique to the SCAT-5, the DANA is a mobile phone-based assessment, which uses a cognitive and psychological test to rapidly identify TBI [18]. Unfortunately, neurophysiological tests like DANA, and other tests that lack a visual component, create a potential for patient motivation to affect results [19,20]. Even the King-Devick test, which measures reading speed, language production and saccades, fails to consider other eye movements that are often impaired by TBI [16,17].

Eye tracking has real potential to fill a void in the world TBI testing. As the only method of objectively and accurately measuring visual behavior, eye tracking can be used to confirm or deny the presence of TBIs in a unique way. The aforementioned objectivity and accuracy address many of the issues with conventional TBI tests, including subjectivity and inaccurate reporting. Smooth pursuits are just one of the metrics that can be measured using eye tracking, and even alone they offer great insight into a patient’s condition. Though there are many studies looking at differences in smooth pursuit between mTBI and control groups, none compare control groups to three different levels of TBI. These data are just as important as mTBI comparison, and it is important that it is studied. Combined with other metrics, smooth pursuits have real potential to offer a quick and easy to administer TBI detection system [31]. The speed and ease of eye-tracking technology could even prove useful in a sports setting, especially in return to play decisions.

### Limitations

The present study had a small number of limitations. First, the study sample size (n = 92) was relatively small, but it was considered adequate for the present studies’ purposes. Ideally, a more far-reaching study could be done that would encompass a greater geographic area and would include more international diversity with which to compare results and identify key trends. Second, the hypothesized significant differentiation of mTBI symptoms compared with healthy controls was not observed. This was disappointing given the incidence, prevalence and economic costs of mTBI. VSP, however, remains a useful indicator in a more holistic assessment of concussion symptoms.

## Conclusion

Although eye tracking represents a great many opportunities, given its infancy as a form of TBI symptom detection, it is recommended that more research should be done to amass a broader sample size for comparison purposes.

**Summary points**

- Early detection and diagnosis are the key to securing positive outcomes for traumatic brain injury (TBI) patients.
- Many tests for TBI are highly subjective, which brings with it a number of limitations, most concerning accuracy of diagnosis. Therefore, there is room for objective measurement tools that can offer a more reliable indicator of brain injury.
- Eye-tracking technology can offer insights into damaged neurocircuitry indicative of TBI.
- To date, horizontal and vertical saccades metrics have been shown to be a simple, quick and accurate measure of TBI that can accurately differentiate between individuals with different levels of severity.
- The present study hypothesized that vertical smooth pursuit eye movements may be a promising addition to a growing group of measures that are active and measure deficits in different brain regions.
- The results indicated that vertical smooth pursuit eye movements measured through eye-tracking technology could accurately differentiate between individuals with moderate and severe levels of TBI.
